# The Influence of Genetic Polymorphic Variability of the Catechol-O-methyltransferase Gene in a Group of Patients with a Diagnosis of Behavioural Addiction, including Personality Traits

**DOI:** 10.3390/genes15030299

**Published:** 2024-02-26

**Authors:** Remigiusz Recław, Krzysztof Chmielowiec, Aleksandra Suchanecka, Agnieszka Boroń, Jolanta Chmielowiec, Aleksandra Strońska-Pluta, Michał Tomasz Kowalski, Jolanta Masiak, Grzegorz Trybek, Anna Grzywacz

**Affiliations:** 1 Foundation Strong in the Spirit, 60 Sienkiewicza St., 90-058 Łódź, Poland; health@mocniwduchu.pl; 2 Department of Hygiene and Epidemiology, Collegium Medicum, University of Zielona Góra, 28 Zyty St., 65-046 Zielona Góra, Poland; chmiele@vp.pl (K.C.); chmiele1@o2.pl (J.C.); 3 Independent Laboratory of Health Promotion, Pomeranian Medical University in Szczecin, Powstańców Wielkopolskich 72 St., 70-111 Szczecin, Poland; aleksandra.suchanecka@pum.edu.pl (A.S.); aleksandra.stronska@pum.edu.pl (A.S.-P.); 4 Department of Clinical and Molecular Biochemistry, Pomeranian Medical University in Szczecin, Aleja Powstańców Wielkopolskich 72 St., 70-111 Szczecin, Poland; agnieszka.boron@pum.edu.pl; 5 Clinical Department of Cardiology, Nowa Sól Multidisciplinary Hospital, 67-100 Nowa Sol, Poland; kowaltmd@wp.pl; 6 II Department of Psychiatry and Psychiatric Rehabilitation, Medical University of Lublin, 1 Głuska St., 20-059 Lublin, Poland; jolantamasiak@wp.pl; 7 Department of Oral Surgery, Pomeranian Medical University in Szczecin, 70-111 Szczecin, Poland; grzegorz.trybek@pum.edu.pl; 8 Maxillofacial Surgery Clinic, 4th Military Clinical Hospital in Wroclaw, ul. Rudolfa Weigla 5, 50-981 Wrolaw, Poland

**Keywords:** behavioral addiction, personality traits, COMT rs4680

## Abstract

Gambling Disorder (GD) is characterised by a harmful, enduring, and recurrent involvement in betting-related behaviours. Therefore, GD shares similar biological mechanisms and symptoms to substance use disorders (SUD). Therefore, in this study, we chose the behavioural addictions group. During the examination and recruitment to the study, it turned out that all the people undergoing treatment for gambling addiction were also addicted to amphetamines, which is consistent with the biological mechanism related to cerebral neurotransmission. The aim of the study was to investigate the association of the *COMT* gene polymorphism with behavioral addiction. The study group consisted of 307 participants: 107 men with gambling disorder and amphetamine dependency (mean age = 27.51, SD = 5.25) and 200 non-addicted, nor dependent, free from neuro-psychiatric disorders control group men (mean age = 20.20, SD = 4.51). Both groups were subjected to psychometric evaluation using the State-Trait Anxiety Inventory and the NEO Five-Factor Personality Inventory. Genomic DNA was extracted from venous blood following standard protocols. Determination of the rs4680 polymorphism in the *COMT* gene was performed using the real-time PCR technique. Statistically significant differences in the frequency of rs4680 genotypes were found in the tested sample of subjects compared with the control group (*p* = 0.03543). Subjects with gambling disorder and amphetamine use disorder compared to the control group obtained higher scores in the assessment of the STAI trait scale (*p* = 0.0019), state scale (*p* < 0.0000), and NEO-FFI Neuroticism scale (*p* < 0.0000). Significantly lower results were obtained for the NEO-FFI Agreeability scale (*p* < 0.0000). Additionally, a significant statistical impact of gambling disorder and amphetamine use disorder, and the *COMT* rs4680 genotype was demonstrated for the score of the STAI trait (*p* = 0.0351) and state (*p* = 0.0343) and the NEO-FFI Conscientiousness scale (*p* = 0.0018). We conclude that *COMT* and its polymorphic variant influence the development of addiction. Still, considering its multifactorial and polygenic nature, it should be combined with other factors such as personality.

## 1. Introduction

Behavioral addictions have an identical neurobiological basis as substance addictions. Gambling Disorder (GD) is characterised by a harmful, enduring, and recurrent involvement in betting-related behaviours [1]. Hence, Gambling Disorder exhibits biological processes and symptoms that are akin to those found in Substance Use Disorders (SUD) [2]. Therefore, in the present study, a homogeneous subgroup of people with behavioural addiction was selected. This choice was dictated by the literature but also by the fact that in rehab facilities, groups of behavioural addicts are formed as separate, homogeneous subgroups. Previous research has shown that GD and SUD often co-occur [3,4], and both of these conditions may be very different in a population with only one of these diagnoses [4]. Specifically, symptoms of SUD and coexisting GD are typically complex [5,6], and treatment can be particularly challenging [7]. The relative time of onset of substance use disorder and gambling disorder is problematic. Specifically, SUD may precede GD in some individuals, whereas it might follow GD in another group, while the co-occurring disorders are more often present before GD [8,9]. Therefore, in this study, we chose the behavioural addictions group. During the examination and recruitment to the study, it turned out that all people undergoing treatment for gambling addiction were also addicted to amphetamines, which is consistent with the biological mechanism related to cerebral neurotransmission. It was also important for us to select a gene related to brain neurotransmission, which was described in detail in the introduction.

Impairments in many post-cognitive areas, including inhibition, working memory, decision-making, cognitive flexibility and executive planning, have been reported in studies regarding adults with gambling problems [10,11]. Dopamine is crucial for cognitive functions that rely on the frontostriatal circuit. It is believed that dopamine imbalances can significantly influence impulsive behaviours, especially those associated with decision-making and inhibitory control [12]. A unique role in the regulation of dopamine in the prefrontal cortex is played by the enzyme catechol-O-methyltransferase (COMT) [13] and is recognised in many psychiatric disorders, particularly those characterised by high impulsivity, as a potential pharmacological target for the treatment of cognitive dysfunction [14,15]. 

As the dopaminergic system undoubtedly influences the development and course of addiction, the present study focused on the gene encoding one of the enzymes involved in the metabolism of dopamine, catechol-o-methyltransferase, which is a postsynaptic enzyme that degrades catecholamines (epinephrine, dopamine and norepinephrine) [16]. In the *COMT* gene, mapped to chromosome 22q11.1-q11.2, with a size of approximately 27 Kbp, up to 345 polymorphisms have been identified. One functional single nucleotide polymorphism (rs4680) is caused by guanine to adenine substitution at codon 158, resulting in a change of valine (Val) to methionine (Met). This polymorphism may affect dopamine levels, particularly in the prefrontal cortex [17]. Carriers of the Val158 allele synthesise a thermostable form of the enzyme [18], with 40% higher brain activity than the Met158 allele at normal body temperature. As these two alleles are additive, heterozygotes show intermediate activity [19]. Higher extracellular dopamine levels in prefrontal cortex areas and better performance in cognitive tasks have been reported for the low-activity Met158 allele [20]. The Val158 allele, on the other hand, has been associated with positive processing of the signals related to aversive stimuli [21]. This polymorphism has been identified as a risk factor for several neuropsychiatric disorders, including substance use and addiction, obsessive-compulsive disorder (OCD) [22] and attention-deficit/hyperactivity disorder (ADHD) [23]. The low-activity *COMT* allele or genotype has been linked to alcohol problems in several studies [24,25,26]. It has also been shown that the highly active *COMT* allele (Val158) is more common in polysubstance users [27] and heroin abusers [28].

In the present study, the gene encoding *COMT* and its functional polymorphic variant were selected due to their connection with the functioning of the dopaminergic system and the possibility of interaction with the environment. Research on this gene suggests that the effect of the *COMT* Val158Met polymorphism on behaviour should be considered in the context of gene-environment interactions rather than a direct effect. Interestingly, carriers of the methionine allele are more susceptible to stress and environmental factors in some studies of general population samples [29]. The methionine allele is associated with increased anxiety, decreased extraversion and decreased novelty seeking [30,31]. Psychostimulants have different effects in people with different *COMT* gene variants. In Val/Val subjects, amphetamine improves PFC function and performance on tasks measuring working memory or attention [32,33]. Met/Met individuals show better PFC function and are reported to have higher baseline PFC dopamine concentrations than Val/Val carriers under normal conditions [34]. However, amphetamine exposure has been shown to impair PFC function, working memory performance and attention in Met/Met carriers [32,33]. The Val158Met substitution has been shown to have sex-specific consequences. In vitro cell studies have shown that physiological levels of 17-β-estradiol can downregulate *COMT* gene transcription and COMT protein expression [35,36]. In another study, an association was found between Met alleles with low levels of activity and obsessive-compulsive disorder in men, but not in women [37]. Studies in mice have shown that homozygous *COMT*-knockout females develop increased anxiety in a light-dark model compared to *COMT*-knockout males. In the same study, heterozygous *COMT* knockout males showed increased aggressive behaviour compared to other male genotypes [38].

The ‘endophenotypic’ approach [39,40,41,42] is a recent conceptual approach that may help reduce the heterogeneity of substance use disorder phenotypes and provide a framework for identifying general and specific factors influencing SUD [39,40,41,42]. Considered genetically ‘simpler’ than SUDs, endophenotypes are measurable traits that lie between the clinical phenotype and the disease susceptibility genotype [39,40,41]. Neurocognitive function is particularly well suited as an endophenotype. It is more objective than self-reported measures. According to researchers, impulsivity has a significant relationship with addiction. The neurocognitive dimensions of impulsivity have received the strongest support as a potential SUD endophenotype among the various neurocognitive functions associated with SUD [40,43,44,45]. Several dimensions characterise neurocognitive impulsivity. These are typically measured using tasks that fall into one of two categories [46]: (1) decision/choice impulsivity, referring to the tendency to choose immediate but smaller rewards over delayed but larger rewards; may involve deficits in delayed gratification and self-control [41], as assessed by decision making tasks involving different risk, reward and delay events [41,47]; (2) motor/action impulsivity, referring to the ability to fail to inhibit inappropriate actions, as assessed by response inhibition tasks [48,49]. People addicted to different classes of drugs, such as opiates and stimulants, may differ significantly in these dimensions of impulsivity [50,51,52,53].

The factor related to impulsivity and other traits also seems important, as described by Boscutti et al. in 2022, considering various genetic factors [54]. Another study supporting our approach to analysis was presented by Fang et al., who examined COMT in a clinical context [55].

In a comprehensive and holistic approach to addiction and dependency as a dysfunction of the dopaminergic system in the brain, personality-related factors cannot be forgotten or omitted. Therefore, in the presented study we analysed personality dimensions measured by the NEO-Five Factor Inventory, and anxiety measured by the State-Trait Anxiety Inventory together with *COMT* rs4680. The aim of the study was to investigate the association of the *COMT* gene polymorphism with behavioral addiction.

We emphasise that this is the first study of its kind to consider the simultaneous analysis of psychological and genetic factors, also taking into account interactions. The study included a group of men as a homogeneous group, not only biologically but also psychologically.

## 2. Materials and Methods

The study group consisted of 307 subjects: 107 men with gambling disorder and amphetamine dependence, during three months of abstinence in an addiction treatment facility (mean age = 27.51, SD = 5.25) and 200 non-addicted, nor dependent, free from neuropsychiatric disorders control group men (mean age = 20.20, SD = 4.51). The study was approved by the Bioethics Committee of the Pomeranian Medical University in Szczecin (KB-0012/106/16 (17 October 2016)). All participants gave written informed consent before participating in the study. The study was conducted in the Independent Laboratory of Health Promotion at the Pomeranian Medical University in Szczecin.

### 2.1. Psychometric Tests

In the study group and in the control group, the NEO-FFI personality test and the State-Trait Anxiety Inventory (STAI) were performed. NEO-FFI defines five main traits—extraversion, openness, conscientiousness, agreeableness and neuroticism. The STAI inventory, on the other hand, describes anxiety as a trait and/or as a state [56]. A psychologist interpreted the results of the psychometric tests. The results were converted to the sten scale. The interpretation included Polish standards for adults, which assume a meagre rating for sten 1–2, a low rating for sten 3–4, an average rating for sten 5–6, a high rating for sten 7–8 and a very high rating for sten 9–10.

The MINI International Neuropsychiatric Interview was used to evaluate the eligibility for inclusion into the control group [57]. It is a structured diagnostic interview assessing mental disorders. Mini focuses on the patient’s current condition. Past diagnoses are analysed to determine if they are clinically significant for the present.

### 2.2. Genotyping

Standard procedures were used to isolate genomic DNA from venous blood.

The isolation of genetic material was carried out according to ROCHE standards and procedures. The selection of reagents and primers can be found in the description of the ROCHE real-time PCR methodology.

Determination of the rs4680 polymorphism in the *COMT* gene was performed using the real-time PCR technique. Melting curves were generated for each sample by plotting the fluorescence signal as a function of temperature. The peaks of the *COMT* rs4680 polymorphic site were read at 53.29 °C for the A allele and at 59.93 °C for the G allele.

### 2.3. Statistical Analysis

The HWE software was used to test the concordance of the alleles frequency distribution with the Hardy–Weinberg equilibrium (https://wpcalc.com/en/equilibrium-hardy-weinberg/ (accessed on 3 December 2023).

A multivariate analysis of factor effects ANOVA was used to analyse the relations between *COMT* rs4680 variants, gambling disorder and amphetamine dependency, and control subjects, as well as the NEO Five-Factor Inventory [NEO-FFI/scale STAI/ × genetic feature × control and gambling disorder and amphetamine dependency × (genetic feature × control and gambling disorder and amphetamine dependency)]. The homogeneity of variance condition was met (Levene test *p* > 0.05). The variables under analysis did not follow a normal distribution. The U Mann–Whitney test was used to compare sten scores for the NEO Five-Factor Inventory (Neuroticism, Extraversion, Openness, Agreeableness, and Conscientiousness). The association of the *COMT* rs4680 genotype and alleles in both groups was tested using the chi-squared test (*n* = 307, φ = 0.15; α = 0.05; statistical power 0.646). The computations were performed using STATISTICA 13 (Tibco Software Inc., Palo Alto, CA, USA) for Windows (Microsoft Corporation, Redmond, WA, USA).

## 3. Results

The frequency distributions were consistent with the Hardy–Weinberg equilibrium (HWE) in both the group with a gambling disorder and amphetamine use disorder, as well as the control group (Table 1).

Statistically significant differences in the frequency of *COMT* rs4680 genotypes were found in the tested sample of subjects with gambling disorder and amphetamine use disorder compared with the control group (G/A 0.57 vs. G/A 0.42; A/A 0.24 vs. A/A 0.36; G/G 0.19 vs. G/G 0.22, χ^2^ = 6.681, *p* = 0.03543). No statistically significant differences in the frequency of *COMT* rs4680 alleles were found between subjects with gambling disorder and amphetamine use disorder and the control group (A 0.53 vs. A 0.57; G 0.47 vs. G 0.43, χ^2^ = 0.990, *p* = 0.3187) (Table 2).

The means and standard deviations of the NEO-FFI and STAI state and trait scores for subjects with gambling and amphetamine use disorders and controls are shown in Table 3.

Subjects with gambling disorder and amphetamine use disorder compared to the control group obtained higher scores in the assessment of STAI trait scale (6.98 vs. 5.33; Z = 3.106; *p* = 0.0019), and state scale (5.60 vs. 4.77; Z = 5.575; *p* < 0.0000), and NEO-FFI Neuroticism scale (6.58 vs. 4.76; Z = 6.657; *p* < 0.0000). Significantly lower results were obtained for the NEO-FFI Agreeability scale (4.28 vs. 5.54; Z = −4.941; *p* < 0.0000).

**Figure 1:**
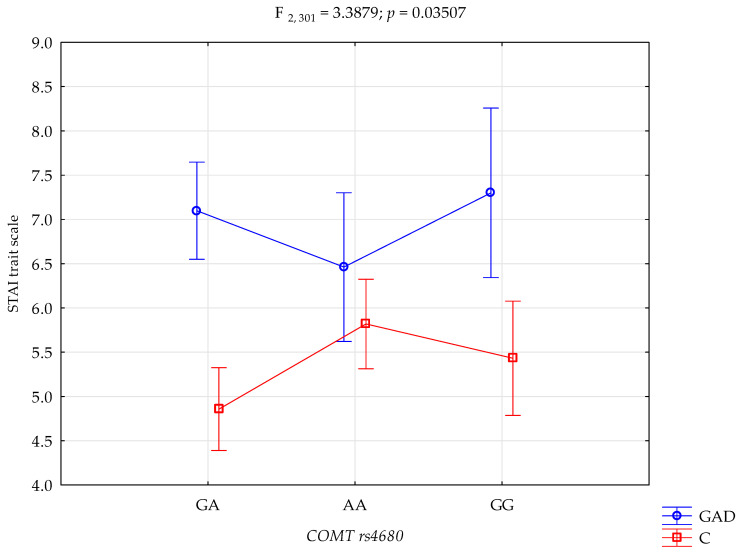
Interaction between gambling disorder and amphetamine use disorder (GAD); *n* = 107/control (C), and *COMT* rs4680, and STAI trait scale.

**Figure 2:**
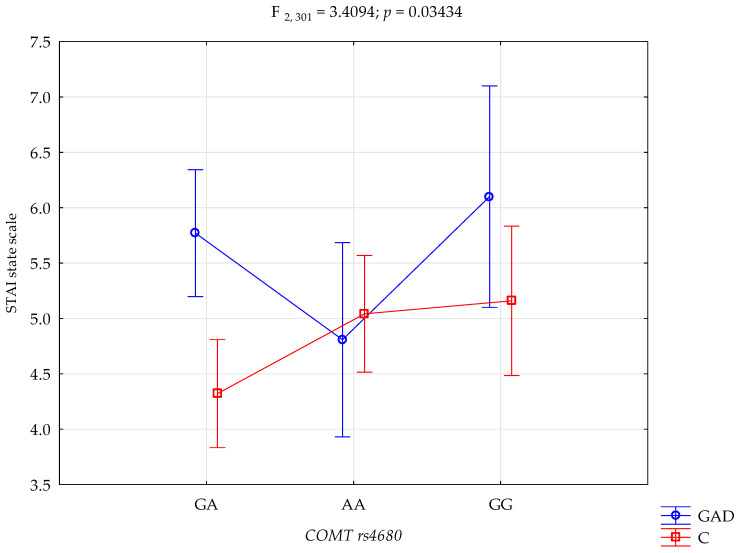
Interaction between gambling disorder and amphetamine use disorder (GAD); *n* = 107/control (C), and *COMT* rs4680, and STAI state scale.

The results of the 2 × 3 factorial ANOVA of the NEO Five-Factor Personality Inventory and the State-Trait Anxiety Inventory sten scales are summarised in Table 4. The significant statistical impact of gambling disorder and amphetamine use disorder and the *COMT* rs4680 genotype was demonstrated for the score of the STAI trait scale. There was a statistically significant effect of the *COMT* rs4680 genotype interaction and gambling disorder and amphetamine use disorder or not using (control group), on the STAI trait scale (F_2,301_ = 3.39; *p* = 0.0351; η^2^ = 0.022; Figure 1). The power observed for this factor was 64%, and approximately 2% was explained by the polymorphism of *COMT* rs4680 and gambling disorder and amphetamine use disorder, or lack thereof, on STAI trait score variance. There was also a statistically significant effect of gambling disorder and amphetamine use disorder or the control group on the STAI state scale score (F_2,301_ = 3.41; *p* = 0.0343; η^2^ = 0.022; Figure 2). The power observed for this factor was 64%, and approximately 2% was explained by the polymorphism of *COMT* rs4680 and gambling disorder and amphetamine use disorder, or lack thereof, on the variance in the STAI state scale score.

**Figure 3:**
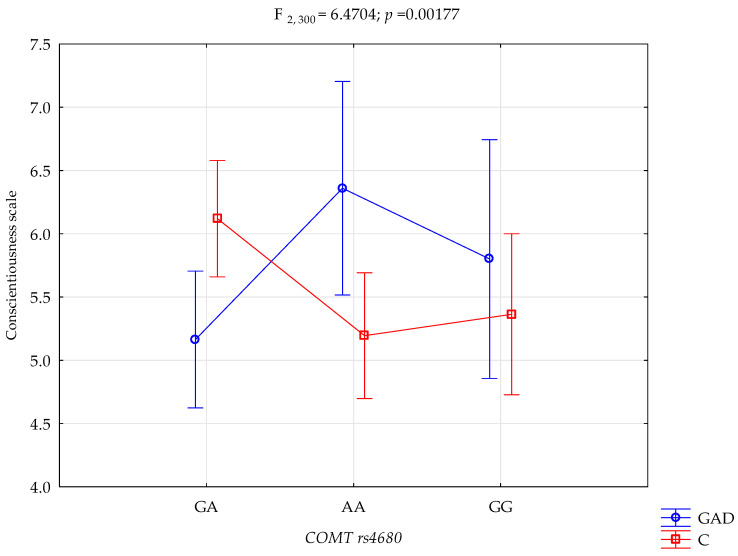
Interaction between gambling disorder and amphetamine use disorder (GAD)/control (C) and *COMT* rs4680 and Conscientiousness scale.

A significant statistical impact of gambling disorder and amphetamine use disorder and the *COMT* rs4680 genotype was demonstrated for the score of the NEO-FFI Conscientiousness scale. There was a statistically significant effect of the *COMT* rs4680 genotype interaction and gambling disorder and amphetamine use disorder or not using (control group) on the Conscientiousness scale (F_2,301_ = 6.47; *p* = 0.0018; η^2^ = 0.041; Figure 3). The power observed for this factor was 90%, and approximately 4% was explained by the polymorphism of *COMT* rs4680, gambling disorder and amphetamine use disorder, or lack thereof, on the trait of the Conscientiousness score variance. Table 5 shows the results of the post hoc test.

There is a significant interaction between gambling disorder and amphetamine use disorder, and the *COMT* gene rs4680 polymorphism in the outcome score level of anxiety as a trait. Subjects with gambling disorder and amphetamine use disorder with the GA genotype have significantly higher levels of anxiety as a trait compared to the control group with the GA, AA and GG genotypes. Similarly, gambling disorder and amphetamine use disorder subjects with the GG genotype have significantly higher levels of anxiety as a trait compared to the control group with the GA, AA and GG genotypes. Subjects with gambling disorder and amphetamine use disorder with the AA genotype have significantly higher levels of anxiety as a trait compared to the control group with the GA genotype. The control group with the GA genotype has significantly lower anxiety as a trait compared to the control group with the AA genotype (Table 5).

There is a significant interaction between gambling disorder and amphetamine use disorder, and the *COMT* gene rs4680 polymorphism in the outcome score level of anxiety as a state. Gambling disorder and amphetamine use disorder subjects with the GA genotype have significantly higher levels of anxiety as a state compared to the control group with the GA genotype. Gambling disorder and amphetamine use disorder subjects with the GG genotype have significantly higher levels of anxiety as a state compared to the control group with the GA genotype The control group with the GA genotype has significantly lower levels of anxiety as a state compared to the control group with the AA and GG genotype (Table 5).

There is a significant interaction between gambling disorder, amphetamine use disorder, and *COMT* gene polymorphism in the outcome of Conscientiousness level. Gambling disorder and amphetamine use disorder subjects with the GA genotype have significantly lower scores of Conscientiousness compared to the control group with the GA genotype. Conversely, gambling disorder and amphetamine use disorder subjects with the AA genotype have significantly higher scores of Conscientiousness compared to the control group with the AA genotype. Furthermore, Gambling disorder and amphetamine use disorder subjects with the GA genotype had significantly lower scores of Conscientiousness compared to the Gambling disorder and amphetamine use disorder subjects with the AA genotype. Also, the control group with the GA genotype has significantly higher scores of Conscientiousness compared to the control group with the AA genotype (Table 5).

## 4. Discussion

The aim of the presented study was the case-control analysis of over one hundred male subjects with amphetamine use disorder and gambling disorder. We analysed the catechol-o-methyl transferase single nucleotide polymorphism rs4680; personality was measured with the NEO-FFI inventory and anxiety was measured as a state and trait by the STAI questionnaire. Additionally, we analysed the interactions between the *COMT* genotypes, personality traits and anxiety measures. The main findings of the analyses are as follows: the GA genotype is statistically significantly more frequent in the study group compared to the control group; the study group had higher scores on the anxiety as a trait and as a state scale and higher scores on the neuroticism scale compared to the control group and lower scores on the agreeableness scale. We also showed an interaction between anxiety as a trait and the rs4680 genotypes of the *COMT* gene and conscientiousness and the rs4680 genotypes of the *COMT* gene.

In the presented study, we analysed subjects burdened with both behavioural addiction, gambling disorder and substance use disorder, amphetamines. A high degree of concordance between substance use disorders and other potentially addictive behaviours has been demonstrated in epidemiological studies [48,58,59,60,61,62,63]. There also appears to be an overlap in the psychological mechanisms at the basis of these behaviours. Specific personality traits [49], e.g., impulsivity [64], and motivational factors [65] appear to play an important role in both substance use and other potential behavioural addictions. Research also suggests a strong neurobiological link between substance use disorders and behavioural addictions from biochemical, neuroimaging, genetic and treatment perspectives [66,67,68,69]. Individuals with those disorders derive pleasure, stimulation and satisfaction from their impulsive behaviour (e.g., gambling addiction, compulsive shopping). In addition, a common psychological and molecular pathway underlying impulsive, compulsive and addictive behaviours is suggested by the Reward Deficiency Syndrome hypothesis [70]. It is often the case that methamphetamine use disorder and gambling disorder cooccur. People with both of these disorders tend to be more difficult to treat than people with only one of these disorders [9]. Compared to non-gamblers, adolescent gamblers were more likely to drink alcohol, smoke tobacco and use illicit drugs in an earlier study [62]. Similarly, the study of a sample of young people found that men who were nicotine, alcohol or cannabis users were almost twice as likely to be problem gamblers than those who were not [48]. In another study [58], it was found that people with alcohol use disorders had significantly higher scores on scales for gambling disorder, compulsive buying and sex addiction when compared to control subjects. Higher levels of impulsivity and alcohol craving were also found in people with alcohol use disorder and co-occurring behavioural addictions. The main findings of this study suggest that there is an association between the use of certain substances (in particular, the regular use of alcohol) and the severity of certain potentially addictive behaviours. In addition, some potentially addictive behaviours (problematic internet use, gambling and eating disorders) appear to be more related to substance use than others (e.g., hair pulling), suggesting that addictions may be divided into different homogeneous subgroups [71].

In our study, we analysed only male subjects since we did not encounter female sub-jects with both gambling disorder and amphetamine use disorder, as both of these disorders are far more frequent in males than in females. Knowing its sex-dependent action, we chose the *COMT* gene for the analysis. The selection of the group was also justified by the analysis of the group of men as a homogeneous subgroup of addicts. This research model is justified due to psychological factors and the course of addiction. 

The effect of COMT on sex may result from a number of possible mechanisms. In both men and women, the Met allele is associated with lower levels of COMT enzyme activity (relative to Val/Val). However, women have lower levels of COMT enzyme activity in the dorsolateral PFC [72] and blood [19] for each genotype (Val/Val, Val/Met, Met/Met) compared to men. Estrogen regulation of COMT may also be a contributor to its sex-specific effects [73]. In addition, significant sex differences have been shown in the dopaminergic systems affected by COMT, which are associated with smoking and addiction more generally [74,75,76]. Functional neuroimaging studies indicate that in contrast to men, women have higher basal synaptic levels of dopamine [77] and may show lower amphetamine-induced dopamine release in the striatum [78]. In smokers, the smoking-induced striatal dopamine release regional location differs between sexes as well, i.e., in men we observed increased activation of the ventral striatum, and in women of the dorsal striatum [79]. Women have been shown to experience a greater decrease in dopamine in the nucleus accumbens following nicotine withdrawal [80]. Additionally, studies have shown sex differences in the cognitive impact of dopaminergic interventions [81,82]. Furthermore, the sex-specific effects of the *COMT* genotype on cortical development and morphology have been documented [83,84,85]. The psychiatric phenotypes affected by the *COMT* genotype exhibit sex-specificity [73], including smoking behavior, depression, and anxiety-related phenotypes where we observe a stronger association with the Val allele in women [86,87,88,89].

Our analysis began with an examination of the frequencies of genotypes and alleles of *COMT* rs4680. Statistically significant differences were found in the frequencies of genotypes in the tested sample of subjects with gambling disorder and amphetamine use disorder compared to the control group. The GA genotype was more frequent in cases, and the AA genotype was more frequent in controls. For the alleles, we did not find significant differences. Chmielowiec et al. [90] found no statistically significant differences under the co-dominant model of genotype frequencies for rs4680 in their study regarding patients diagnosed with other-than-cocaine stimulant dependence. Allelic frequencies were also not statistically significant. Zhang et al. [91], whose study showed reduced prefrontal fractional anisotropy only in Met/Met homozygotes who were also drug users, found a significant genotype×drug use status interaction. These data suggest that Met/Met homozygotes may have an increased susceptibility to white matter structural alterations in the context of addiction, which may contribute to previously identified structural and functional prefrontal cortical deficits in addiction.

The personality and anxiety measures were the second analysis we conducted. Subjects with amphetamine use disorder and gambling disorder scored higher on the STAI trait and state scales and the NEO-FFI Neuroticism scale compared to the control group. Significantly lower scores were obtained on the NEO-FFI Agreeableness scale. While comparing the controls and the group of patients with a diagnosis of other-than-cocaine stimulants dependence, for the latter, Chmielowiec et al. [90] observed significantly higher scores on the STAI trait and state scale, and the NEO Five-Factor Inventory scale of Neuroticism and Openness. The study group had significantly lower results on the NEO Five-Factor Inventory scale of Extraversion, Agreeability, and Conscientiousness than the control group. More than half (60%) of participants were classified as having moderate or severe anxiety and/or depression in a study of correlates of anxiety and depression in people who smoke methamphetamine. In multivariate models, being in poor/very poor health, being dependent on methamphetamine and being unemployed were associated with higher odds of both moderate to severe depression and moderate to severe anxiety. Lower odds of moderate or severe depression were associated with living in a large rural town, identifying as Aboriginal and Torres Strait Islander and smoking methamphetamine. Higher odds of moderate or severe anxiety were associated with being female [92]. Anxious people may gamble to cope with negative effects, according to stress reduction theory. It is important to examine moderators, as the literature shows mixed associations between anxiety and gambling behaviour. The research investigated how impulsivity moderates anxiety and problem gambling, as well as gambling, to cope. Since sex differences are important, the moderation of impulsivity has been examined across sexes. Results showed that at both high and low levels of impulsivity, men with higher levels of anxiety scored higher on coping motives for gambling. However, the effect size was larger for men with high impulsivity. Women did not show this moderating effect [93].

The third and final step of the presented study was the interaction analysis. A significant statistical effect of gambling disorder and amphetamine use disorder, and the *COMT* rs4680 genotype was shown for the score on the STAI trait scale. Compared to controls with the GA genotype, dependent subjects with the GA genotype have significantly higher levels of anxiety as a trait. Similarly, compared to the control group with the GG genotype, dependent subjects with the GG genotype have significantly higher levels of anxiety as a trait. There was also a statistically significant effect between gambling disorder and amphetamine use disorder and the control group on the STAI state scale score. Compared to the control group with the GA genotype, people with an addiction with the GA genotype have a significantly higher level of anxiety as a state. For the NEO-FFI Conscientiousness scale score, a significant statistical effect of gambling disorder and amphetamine dependence and the *COMT* rs4680 genotype was demonstrated. Compared to the control group with the GA genotype, dependent subjects with the GA genotype have significantly lower conscientiousness scores. Conversely, compared to the control group with the AA genotype, dependent subjects with the AA genotype have significantly higher Conscientiousness scores. The analysis of the interactions between dependency on other-than-cocaine stimulants and *COMT* rs4680, the STAI trait scale, the STAI state scale, the NEO-FFI neuroticism scale and the NEO-FFI extraversion scale showed significant results. The G/G *COMT* rs4680 genotype polymorphism was associated with higher STAI trait and STAI state scores in patients dependent on other stimulants. However, there were no such interactions in the control group, suggesting that hypodopaminergic activity in these patients may more likely be a COMT function [90].

## 5. Conclusions

In the presented study, we see that addictions should be analysed multi-factorially. We can conclude that *COMT* and its polymorphic variant influence the development of addiction. Still, considering its multifactorial and polygenic nature, it should be combined with other factors such as personality. The presented group is also interesting, as it confirms the multithreadedness and combination of behavioural addiction with substance addiction. We hope that these, and similar discoveries, will translate into clinical practice in the future.

There are also limitations to the study. A similar analysis scheme should be carried out on a larger group of subjects and taking into account a larger number of tested genes.

## Figures and Tables

**Table 1:** Hardy-Weinberg’s equilibrium for the *COMT* rs4680 polymorphism.

Hardy-Weinberg Equilibrium including Analysis for Ascertainment Bias		Observed (Expected)	Allele Freq	χ^2^ (*p* Value)
*COMT* rs4680 Subjects with gambling disorder and amphetamine use disorder *n* = 107	G/A	26 (29.8)	p (ins) = 0.47 q (del) = 0.53	2.212 (0.137)
	A/A	61 (53.3)		
	G/G	20 (23.8)		
*COMT* rs4680 Control *n* = 200	G/A	72 (66.1)	p (ins) = 0.43 q (del) = 0.57	2.889 (0.0891)
	A/A	86 (97.8)		
	G/G	42 (36.1)		

*p*—statistical significance, χ^2^ test.

**Table 2:** Frequency of the genotypes and alleles of the *COMT* rs4680 polymorphism in the subjects with gambling disorder and amphetamine use disorder and control subjects.

	*COMT* rs4680				
	Genotypes			Alleles	
	G/A *n* (%)	A/A *n* (%)	G/G *n* (%)	A *n* (%)	G *n* (%)
Subjects with gambling disorder and amphetamine use disorder *n* = 107	61 (57.01%)	26 (24.30%)	20 (18.69%)	113 (52.80%)	101 (47.20%)
Control *n* = 200	86 (42.00%)	72 (36.00%)	42 (22.00%)	228 (57.00%)	172 (43.0%)
χ^2^ (*p* value)	6.681 (0.03543) *			0.990 (0.3187)	

*n*—number of subjects. *—significant statistical differences.

**Table 3:** STAI and NEO Five-Factor Inventory sten scores in subjects with gambling disorder and amphetamine use disorder, and controls.

STAI/NEO Five-Factor Inventory	Subjects with Gambling Disorder and Amphetamine Use Disorder M ± SD (*n* = 107)	Control M ± SD (*n* = 200)	Z	(*p*-Value)
STAI trait scale	6.98 ± 2.30	5.33 ± 2.14	3.106	0.0019 *
STAI state scale	5.60 ± 2.61	4.77 ± 2.11	5.575	0.0000 *
Neuroticism scale	6.58 ± 2.28	4.76 ± 1.94	6.657	0.0000 *
Extraversion scale	5.99 ± 2.18	6.28 ± 2.00	−1.143	0.2529
Openness scale	4.77 ± 2.02	4.56 ± 1.64	0.765	0.4442
Agreeability scale	4.28 ± 1.82	5.54 ± 2.04	−4.941	0.0000 *
Conscientiousness scale	5.57 ± 2.24	5.62 ± 2.15	0.119	0.9054

*p*, statistical significance with Mann–Whitney U-test; *n*, number of subjects; M ± SD, mean ± standard deviation; * statistically significant differences.

**Table 4:** The results of 2 × 3 factorial ANOVA for gambling and amphetamine use disorder subjects and controls, NEO-FFI, STAI and *COMT* rs4680.

STAI/NEO Five-Factor Inventory	Group	*COMT* rs4680				ANOVA		
		G/A *n* = 145 M ± SD	A/A *n* = 98 M ± SD	G/G *n* = 64 M ± SD	Factor	F (*p* Value)	η^2^	Power (alfa = 0.05)
STAI trait scale	Gambling disorder and amphetamine use disorder subjects (GAD); *n* = 107	7.10 ± 2.28	6.46 ± 2.27	7.30 ± 2.43	intercept GAD/C *COMT* rs4680 GAD/C × *COMT* rs4680	F_1,301_ = 1881.08 (*p* < 0.0001) F_1,301_ = 31.07 (*p <* 0.0001) * F_2,301_ = 0.64 (*p* = 0.5245) F_2,301_ = 3.39 (*p* = 0.0351) *	0.862 0.094 0.004 0.022	1.000 1.000 0.158 0.636
	Control (C); *n* = 200	4.86 ± 2.05	5.82 ± 2.06	5.43 ± 2.29				
STAI state scale	Gambling disorder and amphetamine use disorder subjects (GAD); *n* = 107	5.77 ± 2.57	4.81 ± 2.81	6.10 ± 2.36	intercept GAD/C *COMT* rs4680 GAD/C × *COMT* rs4680	F_1,301_ = 1229.59 (*p* < 0.0001) F_1,301_ = 5.87 (*p* = 0.0160) * F_2,301_ = 1.74 (*p* = 0.1775) F_2,301_ = 3.41 (*p* = 0.0343) *	0.803 0.019 0.011 0.022	1.000 0.676 0.363 0.639
	Control (C); *n* = 200	4.32 ± 1.94	5.04 ± 2.17	5.16 ± 2.21				
Neuroticism scale	Gambling disorder and amphetamine use disorder subjects (GAD); *n* = 107	6.72 ± 2.31	6.16 ± 2.32	6.65 ± 2.21	intercept GAD/C *COMT* rs4680 GAD/C × *COMT* rs4680	F_1,301_ = 1739.99 (*p* < 0.0001) F_1,301_ = 41.00 (*p* < 0.0001) * F_2,301_ = 0.09 (*p* = 0.9103) F_2,301_ = 2.12 (*p* = 0.1216)	0.853 0.120 0.001 0.014	1.000 1.000 0.064 0.434
	Control (C); *n* = 200	4.44 ± 1.92	5.10 ± 1.76	4.79 ± 2.21				
Extraversion scale	Gambling disorder and amphetamine use disorder subjects (GAD); *n* = 107	6.03 ± 2.21	6.28 ± 2.17	5.50 ± 2.16	intercept GAD/C *COMT* rs4680 GAD/C × *COMT* rs4680	F_1,301_ = 2029.80 (*p* < 0.0001) F_1,301_ = 1.29 (*p* = 0.2577) F_2,301_ = 1.27 (*p* = 0.2833) F_2,301_ = 1.21 (*p* = 0.2992)	0.871 0.004 0.008 0.008	1.000 0.204 0.274 0.264
	Control (C); *n* = 200	6.62 ± 2.11	6.00 ± 1.90	6.11 ± 1.89				
Openness scale	Gambling disorder and amphetamine use disorder subjects (GAD); *n* = 107	4.82 ± 2.12	4.88 ± 2.15	4.50 ± 1.57	intercept GAD/C *COMT* rs4680 GAD/C × *COMT* rs4680	F_1,301_ = 1558.87 (*p* < 0.0001) F_1,301_ = 0.88 (*p* = 0.3489) F_2,301_ = 0.95 (*p* = 0.3893) F_2,301_ = 0.12 (*p* = 0.8881)	0.839 0.003 0.006 0.001	1.000 0.155 0.213 0.068
	Control (C); *n* = 200	4.73 ± 1.69	4.54 ± 1.53	4.27 ± 1.70				
Agreeability scale	Gambling disorder and amphetamine use disorder subjects (GAD); *n* = 107	4.03 ± 1.79	4.76 ± 1.88	4.45 ± 1.85	intercept GAD/C *COMT* rs4680 GAD/C × *COMT* rs4680	F_1,301_ = 1471.13 (*p* < 0.0001) F_1,301_ = 18.70 (*p <* 0.0001) * F_2,301_ = 0.72 (*p* = 0.4854) F_2,301_ = 1.01 (*p* = 0.3648)	0.831 0.059 0.005 0.007	1.000 0.991 0.172 0.226
	Control (C); *n* = 200	5.57 ± 2.04	5.51 ± 2.12	5.52 ± 1.97				
Conscientiousness scale	Gambling disorder and amphetamine use disorder subjects (GAD); *n* = 107	5.16 ± 2.25	6.36 ± 2.23	5.80 ± 1.99	intercept GAD/C *COMT* rs4680 GAD/C × *COMT* rs4680	F_1,301_ = 1622.79 (*p* < 0.0001) F_1,301_ = 0.59 (*p* = 0.4441) F_2,301_ = 0.15 (*p* = 0.1539) F_2,301_ = 6.47 (*p* = 0.0018) *	0.844 0.002 0.001 0.041	1.000 0.119 0.073 0.904
	Control (C); *n* = 200	6.12 ± 2.21	5.19 ± 1.93	5.36 ± 2.21				

*—significant result; GAD—Gambling disorder and amphetamine use disorder; M ± SD—mean ± standard deviation.

**Table 5:** Post hoc test (Bonferroni) analysis of interactions between gambling disorder and amphetamine use disorder, control and COMT rs4680 and Conscientiousness scale, anxiety as a state and as a trait.

***COMT*** **rs4680 and STAI State Scale**						
	**{1}** **M = 5.77**	**{2}** **M = 4.81**	**{3}** **M = 6.10**	**{4}** **M = 4.32**	**{5}** **M = 5.04**	**{6}** **M = 5.15**
Gambling disorder and amphetamine use disorder G/A {1}		0.0714	0.5739	0.0002 *	0.0662	0.1747
Gambling disorder and amphetamine use disorder A/A {2}			0.0568	0.3410	0.6530	0.5323
Gambling disorder and amphetamine use disorder G/G {3}				0.0018 *	0.0663	0.1257
Control G/A {4}					0.0493 *	0.0485 *
Control A/A {5}						0.7873
Control G/G {6}						
***COMT* rs4680 and STAI Trait Scale**						
	**{1}** **M = 7.10**	**{2}** **M = 6.46**	**{3}** **M = 7.30**	**{4}** **M = 4.86**	**{5}** **M = 5.82**	**{6}** **M = 5.43**
Gambling disorder and amphetamine use disorder G/A {1}		0.2125	0.7194	0.0000 *	0.0008 *	0.0001 *
Gambling disorder and amphetamine use disorder A/A {2}			0.1962	0.0011 *	0.1982	0.0567
Gambling disorder and amphetamine use disorder G/G {3}				0.0000 *	0.0075 *	0.0016 *
Control G/A {4}					0.0063 *	0.1570
Control A/A {5}						0.3527
Control G/G {6}						
***COMT* rs4680 and Conscientiousness Scale**						
	**{1}** **M = 5.16**	**{2}** **M = 6.36**	**{3}** **M = 5.80**	**{4}** **M = 6.12**	**{5}** **M = 5.19**	**{6}** **M = 5.36**
Gambling disorder and amphetamine use disorder G/A {1}		0.0195 *	0.2506	0.0085 *	0.9349	0.6381
Gambling disorder and amphetamine use disorder A/A {2}			0.3847	0.6222	0.0199 *	0.0645
Gambling disorder and amphetamine use disorder G/G {3}				0.5503	0.2648	0.4511
Control G/A {4}					0.0077 *	0.0593
Control A/A {5}						0.6804
Control G/G {6}						

*—significant statistical differences, M—mean.

## Data Availability

The data presented in this study are available on request from the corresponding author. The data are not publicly available due to privacy.
